# Localized amyloidosis of the upper gingiva: a case report

**DOI:** 10.1186/1752-1947-8-198

**Published:** 2014-06-17

**Authors:** Tommaso Bucci, Eduardo Bucci, Ana Maria Puig Rullan, Paolo Bucci, Paolo Nuzzolo

**Affiliations:** 1 Hospital Joan XXIII, C/ Dr. Mallafrè Guasch, 4, 43005 Tarragona, Spain; 2 Universiy Federico II, Via S. Pansini, 5 80131 Naples, Italy

**Keywords:** Oral amyloidosis, Surgical resection, Upper gingiva

## Abstract

**Introduction:**

Localized amyloidosis in the head and neck is a rare and generally benign condition. In the oral cavity, amyloidosis usually involves the tongue or buccal mucosa. We present the second case of oral amyloidosis arising in the gingiva ever reported, to the best of our knowledge.

**Case presentation:**

A 73-year-old White Spanish man presented a persistent nodular mass involving his upper gingiva. The lesion was surgically resected and the histological examination revealed a subepithelial, multinodular amorphous and fibrillar accumulation. Staining of the specimen for Congo red proved positive, exhibiting a reddish colour under light microscopy and apple-green birefringence under polarized light. With immunohistochemical tests, pentagonal amyloid component was demonstrated. An extensive study excluded any systemic involvement; a diagnosis of localized primary amyloidosis was made. After 2 years of follow-up, no clinical progression to systemic amyloidosis or local recurrence was observed.

**Conclusions:**

Localized amyloidosis of the gingiva is an extremely rare condition that seems to show no clinically distinct feature. Histologic examination is the first step towards diagnosis, followed by immunohistochemical tests. The diagnosis of localized amyloidosis should always be integrated with blood tests, a bone marrow biopsy, echocardiography and digestive endoscopy to intercept systemic involvement.

## Introduction

Amyloidosis is a progressive metabolic disease characterized by abnormal deposits of the protein amyloid in one or more organs. The term was coined by Virchow in 1854 in order to describe abnormal extracellular material seen in the liver during an autopsy [1]. The amyloidogenic protein is the basic component of about 25 different protein structures that can misfold and aggregate in insoluble beta-pleated-sheet structures, which are deposited in the extracellular space of different tissues and finally cause functional impairment [2].

This disease may be acquired or hereditary. Also, it can be restricted to a single organ, where the amyloid is both produced and deposited, such as the lungs, the brain, or the skin (localized form) with an excellent prognosis, or may affect many organs of the body, leading to significant morbidity and mortality (systemic form). The mean survival of patients affected by systemic forms is between 5 and 15 months, depending on which parts of the body are affected. Death is commonly caused by kidney failure or arrhythmic episodes. Localized forms have instead an excellent prognosis, in particular when the head and neck district is affected [3].

Amyloidosis can be classified according to its cause: primary, secondary and familial (common in some areas, as Portugal, Japan and Israel). The subtypes of amyloidosis are currently classified on the basis of the specific protein that is deposited, using the prefix A for amyloid, followed by one or more letters that indicate the name of the protein [2]. AL and AA amyloidosis are the most common and known subtypes.

Primary systemic amyloidosis, also known as amyloid light-chain (AL) amyloidosis, is thought to be related with monoclonal gammopathies, in which amyloid-forming immunoglobulin light chains are produced. AL amyloidosis is associated with plasma cell dyscrasia and multiple myeloma; the most commonly affected organs are the kidneys, the liver, the heart and the nerves [2].

Patients with secondary or reactive systemic amyloidosis (AA form) usually have other chronic infectious or inflammatory diseases, such as osteomyelitis, tuberculosis, rheumatoid arthritis, or Crohn's disease. Almost all organs can be affected, most often the kidneys, the liver, and the spleen.

Localized amyloidosis in the head and neck is a rare and generally benign condition. The most common sites of involvement are the thyroid, the larynx and subglottis, whereas in the oral cavity, amyloidosis usually tends to involve the tongue or buccal mucosa [4,5]. We present the second case of oral amyloidosis arising in the gingiva ever reported, to the best of our knowledge [6].

## Case presentation

**Figure 1:**
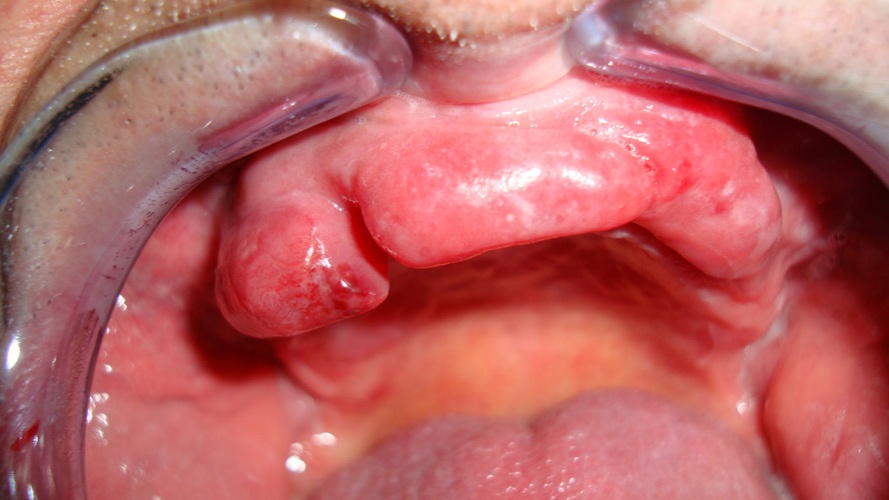
Clinical view of the upper gingiva before surgery.

**Figure 2:**
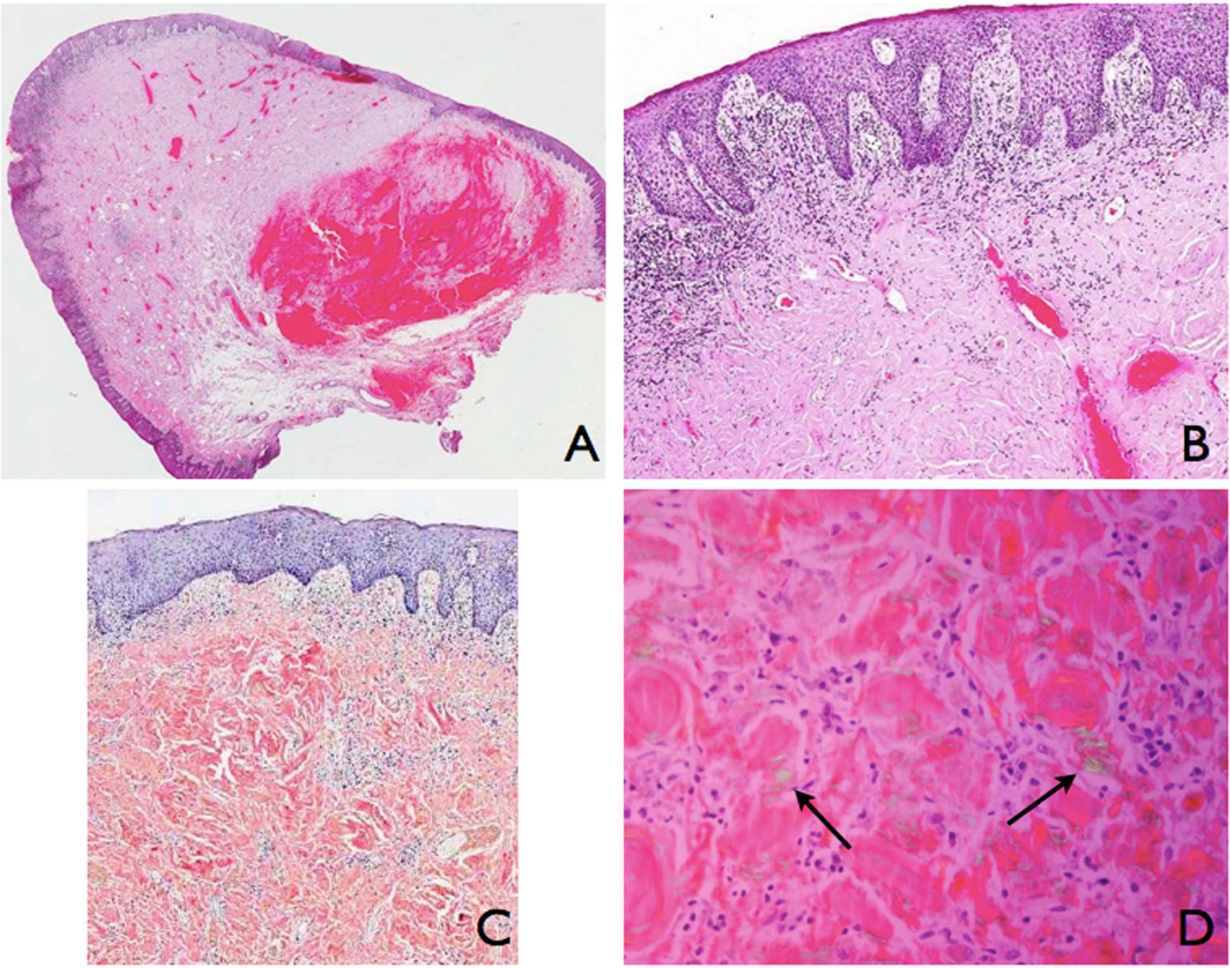
**Histological and immunohistochemical tests. (A)** Panoramic section (haematoxylin and eosin: ×0.6). **(B)** Irregular acanthosis, lymphocytic infiltration and amyloid deposits (haematoxylin and eosin: ×4). **(C)** Congo red stain of amyloid (×10). **(D)** Congo red stain showing characteristic apple-green birefringence (arrows) under polarized light (×60).

**Figure 3:**
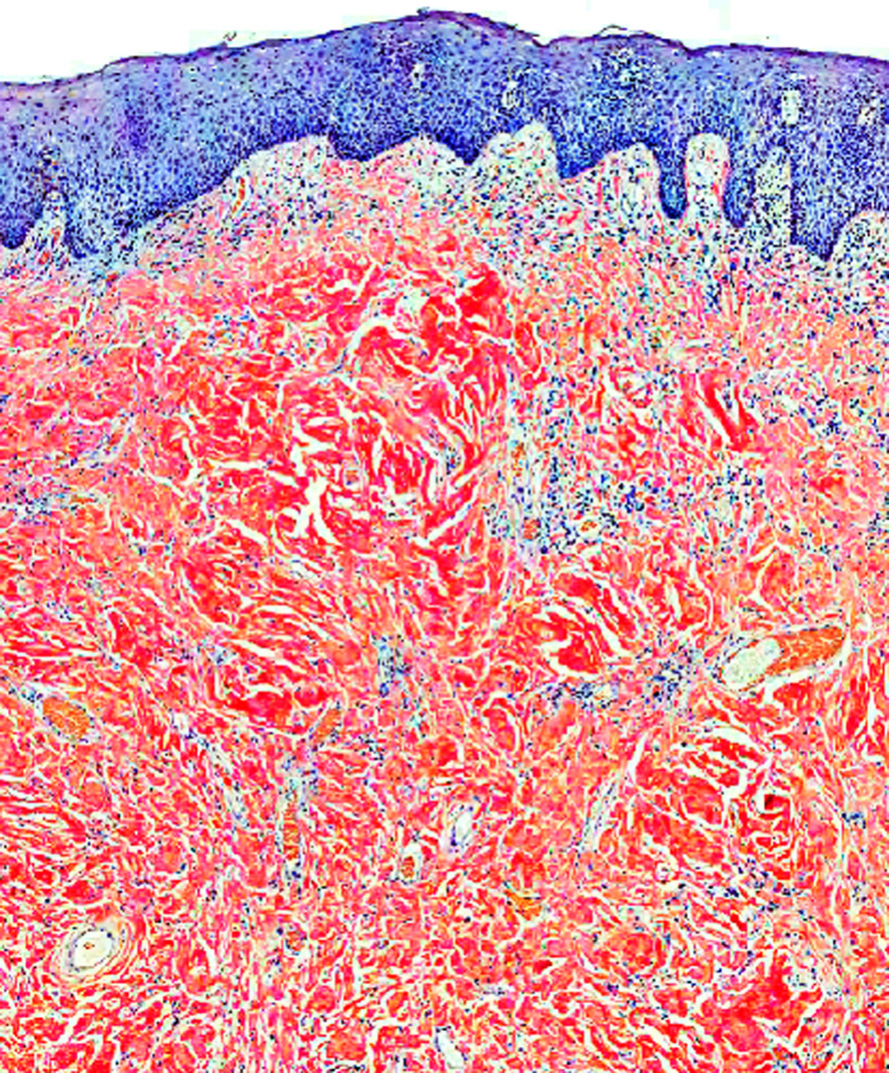
**Congo red staining (×10).** Amyloid material under the mucosal epithelium –detail.

**Figure 4:**
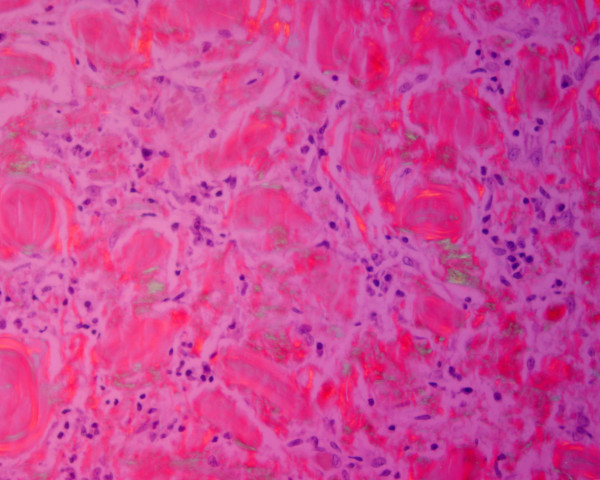
Congo red staining under polarized light (×60) – detail.

A 73-year-old White Spanish man was referred to our Department with a 12-month history of difficulty wearing his full upper denture. Clinical examination revealed a nodular mass involving the upper gingiva that was possibly due to his nonretentive and ill-fitting upper denture (Figure 1).The patient was surgically treated with resection of the whole lesion and primary closure of the surgical wound. Macroscopically, the surgical specimen measured 6cm × 1.4cm × 1.5cm and was a nodular mass covered by normal mucosa. Histological examination showed a subepithelial, multinodular amorphous and fibrillar accumulation (Figures 2A and 2B). The amorphous material had positive staining for Congo red, exhibiting a reddish colour under light microscopy (Figures 2C and 3) and apple-green birefringence under polarized light (Figures 2D and 4). With immunohistochemical tests, pentagonal amyloid component (AP) was demonstrated, whereas the result of the test for AA amyloidosis, with the use of specific monoclonal antibodies, was negative. The patient was submitted to extensive study in order to discover associated systemic diseases or other possible sites of amyloid deposition. Laboratory test results, a bone marrow biopsy, echocardiography and digestive endoscopy excluded any other haematological/immunological disorder or organ dysfunction. From the integration of clinical, laboratory and histological findings, a diagnosis of localized primary amyloidosis was made. After 2 years of follow-up, no clinical progression to systemic amyloidosis or local recurrence was observed.

## Discussion

Amyloidosis is a relatively rare disease, in which localized and systemic forms have differing prognoses, thus requiring different management regimes.

Stem cell transplantation is the gold standard for treatment of primary systemic amyloidosis, but it is not always suitable, depending on the age of the patient, progression of the disease and haematological markers. Combinations of chemotherapy and immunomodulatory agents are an alternative [7,8].

The treatment of localized amyloidosis depends on the organ involved and the grade of functional impairment, being symptomatic most of the time. Follow-up is always required to detect systemic progression [9-13].

Oral localized amyloidosis is not generally associated with systemic amyloidosis and does not usually progress to systemic forms [14].

The AP form of amyloid found in this case has a nonfibrillar structure and represents approximately 10% of the amyloid deposits. It has been demonstrated that AP is an alpha-1 globulin, a normal component of the serum, normally found in vascular basal membranes. It drastically increases in some pathologic conditions as vasculitis, arteriosclerosis, and glomerular nephropathies. None of these conditions has been found in the present case.

Gingival biopsy, in cases in which amyloidosis is suspected, should avoid areas affected by gingivitis and should extend beyond the mucogingival junction, where many fibrous structures are present and where amyloid is more likely to be deposited [4]. Occasionally, gingival biopsy is also preferred, in absence of any gingival lesion, in some cases of suspected systemic amyloidosis where the biopsy of the organs involved is at high risk of morbidity.

After a diagnosis of oral amyloidosis, it is crucial to exclude a systemic involvement. In fact, 25% of the patients with systemic amyloidosis show macroglossia. In this condition, the tongue loses mobility and the patient can lose the ability of retracting it behind the lips. Differential diagnosis with malignant neoplasia must be made considering the absence of ulcerations, pain, bleeding, and adenopathy. In addition, in cases of a carcinoma, the consistency of the tongue is harder [15].

Petechiae, papulae and ulcers can also appear on the oral mucosa, and the involvement of the salivary glands can produce xerostomia [3]. Vascular involvement can produce angiopathy and bleeding [16].

Also, oral amyloidosis can manifest as periodontal destruction and the lesions can be exacerbated by the inflammation of the periodontium [17].

Association between amyloidosis and calcifying odontogenic tumour has also been described [18].

## Conclusions

Local amyloidosis of the gingiva is an extremely rare condition that can present as an unspecific nodular mass. Dentists, oral surgeons, maxillofacial surgeons, pathologists as well as general practitioners should be able to cooperate for the diagnosis, treatment and follow-up. Histologic examination is the first step towards diagnosis, followed by immunohistochemical tests. The diagnosis of localized amyloidosis should always be followed by blood tests, a bone marrow biopsy, echocardiography and digestive endoscopy to intercept systemic amyloidosis or any other haematological/immunological disorder or organ dysfunction.

There is no consensus on the management of local amyloidosis. Surgical treatment of localized forms is indicated to reduce the functional impairment produced by a voluminous mass (for example, a progressive enlargement of the tongue can produce an oropharyngeal blockage, with obstruction of the upper airways). In the present case, we performed surgical excision of the lesion in order to obtain satisfactory retention of the patient’s full upper denture. In any case, follow-up is mandatory, both to manage recurrences of the lesions and, mostly, to monitor the possible evolution of the disease to the systemic form.

## Consent

Written informed consent was obtained from the patient for publication of this case report and accompanying images. A copy of the written consent is available for review by the Editor-in-Chief of this journal.

## Abbreviations

AA: reactive systemic amyloidosis, secondary amyloidosis; AL: light chain amyloidosis, primary systemic amyloidosis; AP: pentagonal amyloid component.

## Competing interests

The authors declare that they have no competing interests.

## Authors’ contributions

TB, EB, PB and PN analysed and interpreted the patient data regarding the lesion, the clinical diagnosis and the exclusion of systemic involvement. TB performed the surgery. PN and TB were major contributors in writing the manuscript. AMPR performed the histological examination and the immunohistochemical tests. The conception and design of the study, the acquisition, analysis and interpretation of data were realized by the authors themselves, as well as the draft of the manuscript and its critical revision. All authors read and approved the final manuscript.
